# Integrating Lithium Sulfide as a Single Ionic Conductor Interphase for Stable All‐Solid‐State Lithium–Sulfur Batteries

**DOI:** 10.1002/advs.202308604

**Published:** 2024-04-23

**Authors:** Xin Wu, Hui Pan, Menghang Zhang, Hanyun Zhong, Zhenjie Zhang, Wei Li, Xinyi Sun, Xiaowei Mu, Shaochun Tang, Ping He, Haoshen Zhou

**Affiliations:** ^1^ Center of Energy Storage Materials & Technology Department of Energy Science and Engineering College of Engineering and Applied Sciences Jiangsu Key Laboratory of Artificial Functional Materials National Laboratory of Solid State Microstructures and Collaborative Innovation Center of Advanced Microstructures Nanjing University Nanjing 210093 P. R. China; ^2^ Department of Materials Science and Engineering College of Engineering and Applied Sciences Nanjing University Nanjing 210093 P. R. China

**Keywords:** all‐solid‐state Li–S battery, interface modification, LGPS, Li_2_S, single ionic conductor

## Abstract

As a very prospective solid‐state electrolyte, Li_10_GeP_2_S_12_ (LGPS) exhibits high ionic conductivity comparable to liquid electrolytes. However, severe self‐decomposition and Li dendrite propagation of LGPS will be triggered due to the thermodynamic incompatibility with Li metal anode. Herein, by adopting a facile chemical vapor deposition method, an artificial solid electrolyte interphase composed of Li_2_S is proposed as a single ionic conductor to promote the interface stability of LGPS toward Li. The good electronic insulation coupled with ionic conduction property of Li_2_S effectively blocks electron transfer from Li to LGPS while enabling smooth passage of Li ions. Meanwhile, the generated Li_2_S layer remains good interface compatibility with LGPS, which is verified by the stable Li‐plating/stripping operation for over 500 h at 0.15 mA cm^−2^. Consequently, the all‐solid‐state Li–S batteries (ASSLSBs) with a Li_2_S layer demonstrate superb capacity retention of 90.8% at 0.2 mA cm^−2^ after 100 cycles. Even at the harsh condition of 90 °C, the cell can deliver a high reversible capacity of 1318.8 mAh g^−1^ with decent capacity retention of 88.6% after 100 cycles. This approach offers a new insight for interface modification between LGPS and Li and the realization of ASSLSBs with stable cycle life.

## Graphical abstract

A thin and homogeneous Li_2_S layer is preconstructed on Li surface as an artificial solid electrolyte interphase to promote the interface stability of LGPS toward Li. With the Li_2_S interlayer, the assembled all‐solid‐state Li–S battery can deliver a high reversible capacity of 1318.8 mAh g^−1^ with greatly improved capacity retention of 88.6% after 100 cycles even at 90 °C.

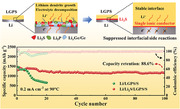

## 1 Introduction

Li–S batteries (LSBs) have received extensive attention and interest because of their distinguished advantages, such as high theoretical energy density (≈2600 W h kg^−1^), environmental friendliness, and low cost.^[^ ^1^, ^2^, ^3^, ^4^ ^]^ However, plagued by the notorious “shuttle effect” (The dissolved polysulfides diffuse to the anode side, causing irreversible loss of active S material and Li corrosion.), liquid LSBs are confronted with poor cycling stability and unsatisfactory energy density.^[^ ^5^, ^6^, ^7^ ^]^ Additionally, safety concerns associated with electrolyte leakage, fire, and explosion also pose a huge challenge to the application of liquid LSBs.^[^ ^8^, ^9^ ^]^

To fundamentally address the above mentioned issues, all‐solid‐state LSBs (ASSLSBs) that replace liquid electrolytes with solid‐state electrolytes (SSEs) have been developed in recent years.^[^ ^10^, ^11^, ^12^, ^13^, ^14^ ^]^ Among several types of SSEs, sulfur‐based SSEs are the most promising ones for ASSLSBs due to their high ionic conductivity, excellent compatibility with sulfur cathodes, and good processability.^[^ ^15^, ^16^, ^17^, ^18^ ^]^

**Figure 1:**
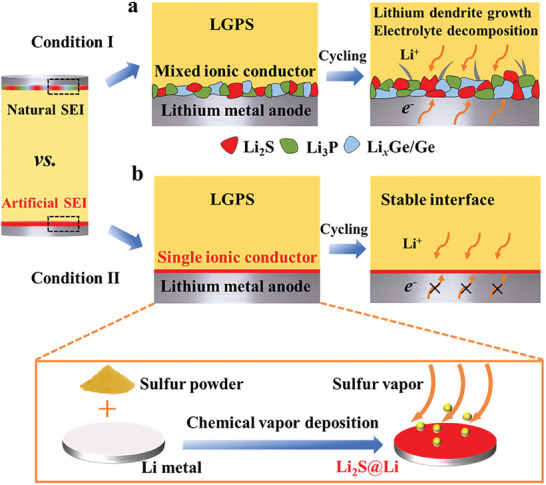
Comparison of naturally formed SEI and artificially constructed SEI between Li and LGPS: a) Schematic of the interface incompatibility between Li and LGPS; b) schematic of the stable interface between Li and LGPS after the introduction of Li_2_S artificial SEI via a chemical vapor deposition method.

As one of the typical and promising sulfur‐based SSEs, Li_10_GeP_2_S_12_ (LGPS) exhibits high ionic conductivity of up to 12 mS cm^−1^ at room temperature, which is comparable to liquid electrolytes and therefore ensures fast Li‐ion migration.^[^ ^19^ ^]^ Nonetheless, restricted by the narrow electrochemical stability window (ESW), LGPS is thermodynamically unstable with Li metal anode.^[^ ^20^, ^21^ ^]^ As demonstrated in **Figure** **1**a, a natural solid electrolyte interphase (SEI) with the mixed ionic conductor property will be spontaneously and randomly generated at the interface once LGPS is in contact with Li. Since the Li*_x_* Ge/Ge components in natural SEI are good electron conductors that enable smooth electron transfer from Li to LGPS, LGPS will be continuously reduced and destroyed during cycling.^[^ ^22^, ^23^, ^24^ ^]^ In addition, the composition distribution varies with different part of the formed SEI, which will also induce uneven Li plating/stripping and ultimately lead to lithium dendrite growth. Therefore, to realize the interface stability between Li and LGPS, several conditions need to be well satisfied. First, the SEI formed at the interface of Li and LGPS should be a single ionic conductor, which allows the free passage of Li ions while electrons are completely blocked. Second, homogeneous composition of the formed SEI is also very critical and should be guaranteed.

Lithium halide (LiX, X = F, I, et al.), which is characterized as a single ionic conductor and is therefore employed as an artificial SEI to enhance the interface stability between Li and electrolyte. For instance, a LiF layer derived from decomposition of liquid electrolyte was introduced to boost the interface compatibility of Li with LGPS. Due to the effective prevention of electron leakage from Li to LGPS, the cycling stability of the assembled ASSLSBs was greatly improved.^[^ ^25^ ^]^ Another research reported that LiI was in situ constructed as an artificial SEI at the Li/LGPS interface. The fabricated LiI layer with unique elongated rice‐shaped structure could maintain intimate contact and suppress interfacial side reactions between Li and LGPS.^[^ ^23^ ^]^

Although lithium halide has good interfacial stability, it should be also pointed out that the formation of LiF layer inevitably involved the use of fluorinated substances as reactants, which is harmful to the environment and uneconomical. And the preparation of LiI layer required a strong oxidizing agent of iodine and is therefore not conducive to large‐scale safety operations. Hence, a more appropriate single ionic conductor that is qualified for an artificial SEI is still needed. In order to obtain a better guidance and screen out potential candidates, the ionic and electronic conductivities of some common SEI components were carefully summarized and presented in Figure S1 (Supporting Information).^[^ ^26^, ^27^, ^28^, ^29^, ^30^, ^31^, ^32^, ^33^, ^34^, ^35^ ^]^ Notably, the lithium sulfide (Li_2_S) demonstrates a good ionic conductivity of ≈10^−5^ S cm^−1^ and poor electronic conductivity of ≈10^−13^ S cm^−1^, which implies that Li ions can easily migrate through Li_2_S while electrons are prohibited.^[^ ^32^, ^36^, ^37^ ^]^ Therefore, it can be speculated that the interface compatibility between Li and LGPS will be achieved after introduction of a Li_2_S artificial SEI (Figure 1b). More importantly, the reactant sulfur utilized in the production of Li_2_S is more favorable for practical applications due to its low cost and chemical mildness. In addition, as one of the components of sulfide‐based electrolytes, Li_2_S for interfacial modification also favors to maintain good stability with both Li and LGPS.

Herein, a thin and uniform Li_2_S layer was in situ constructed on Li surface as an artificial SEI to promote the interface stability with LGPS via a facile chemical vapor deposition (CVD) method. To confirm the successful formation of Li_2_S layer, a series of advanced characterization techniques including X‐ray diffraction (XRD), X‐ray photoelectron spectroscopy (XPS), and transmission electron microscopy (TEM) were applied. Meanwhile, scanning electron microscopy (SEM) and atomic force microscopy (AFM) tests were performed to investigate the morphology and mechanical property of the prepared Li_2_S layer. Then, the role of Li_2_S layer in promoting the interface stability between Li and LGPS and optimizing the electrochemical performance of ASSLSBs were verified. With the robust Li_2_S protective layer, the assembled ASSLSBs of Li/Li_2_S/LGPS/S exhibited excellent reversibility and superb cycling stability. Even at harsh conditions of high S loading and high temperature, decent capacity retentions were still achieved for ASSLSBs, highlighting the feasibility of Li_2_S as an artificial SEI. Such a simple, economical, and environmentally friendly interfacial modification strategy provides an efficient solution to the performance enhancement of ASSLSBs.

## 2 Results and Discussion

### 2.1 Characterization of the Designed Li_2_S Layer

**Figure 2:**
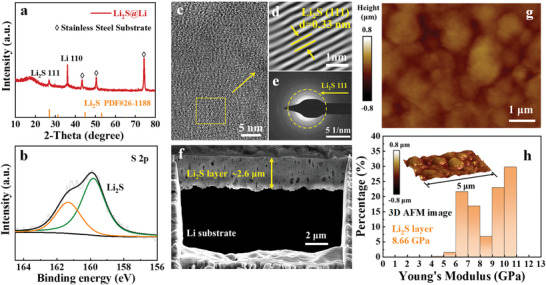
Characterization of the prepared Li_2_S layer: a) XRD pattern of the Li_2_S@Li; b) XPS spectrum of the Li_2_S@Li surface; c) HRTEM image, d) Fourier‐transformed crystalline lattice of the marked area in Figure 2c, and e) SAED image of the Li_2_S scraped from Li surface; f) cross‐sectional focused‐ion beam (FIB) SEM image of the Li_2_S@Li; g) 2D AFM image and h) Young's modulus distribution histogram of the prepared Li_2_S layer and the 3D AFM image showing the topographic feature of Li_2_S layer in the inset.

As illustrated in Figure S2 (Supporting Information), the Li_2_S layer was in situ formed on Li surface through chemical reaction of sulfur vapor with Li (denoted as Li_2_S@Li). XRD experiment was conducted to confirm the successful formation of Li_2_S on Li surface (**Figure** **2**a). The strongest peak appearing at 36.2° belongs to the “110” plane of Li. An additional peak at 27° corresponds to the “111” plane of Li_2_S. And the other three peaks appearing at 43.3°, 50.3°, and 74.3° are attributed to the stainless‐steel substrate. Other than that, no remaining peaks can be observed. To characterize the valence state of elemental S, XPS test was performed for the Li_2_S@Li. As shown in Figure 2b, two peaks located at 159.8 and 161.4 eV in S 2p spectrum refer to the S^2−^, which confirms the presence of Li_2_S.^[^ ^38^ ^]^ The composition of the interlayer was further analyzed by high‐resolution TEM (HRTEM) and selected area electron diffraction (SAED), as displayed in Figure 2c–e. Figure 2d shows the Fourier‐transformed crystalline lattice of the marked area in Figure 2c, and the lattice fringe with interplanar spacing of 0.33 nm corresponds to the “111” plane of Li_2_S, which is further evidenced by the diffraction ring in Figure 2e.

The micro‐morphologies of the pristine Li metal and Li_2_S@Li were investigated by SEM. As shown in Figure S3a,b (Supporting Information), after in situ growth of Li_2_S layer, the smooth Li surface became rough and the silver‐white Li metal turned golden yellow accordingly. To clarify the thickness of the Li_2_S layer, cross‐section observation of the Li_2_S@Li was further conducted by SEM and energy dispersive X‐ray spectroscopy (EDS). Since Li_2_S is electrically insulating, the generation of Li_2_S on Li surface prevents further chemical reactions between sulfur vapor and Li. Such a self‐limiting reaction process ensures that the Li surface can be completely protected by a uniform and dense Li_2_S layer. As displayed in Figure 2f and Figure S3c (Supporting Information), the thickness of the in situ formed Li_2_S layer on Li was determined to be ≈2.6 µm.

AFM test was carried out to analyze the surface roughness and mechanical property of the prepared Li_2_S layer. Figure 2g,h reveals that the generated Li_2_S layer is continuous and relatively flat, with little surface fluctuation. In addition, a high average Young's modulus of 8.66 GPa was achieved for the generated Li_2_S layer (Figure 2h). Studies have demonstrated that Young's modulus higher than 4 GPa is essential for SEIs to avoid Li dendrite penetration. Therefore, the Li_2_S layer with such high Young's modulus could ensure sufficient strength to block the growth of Li dendrites.^[^ ^39^ ^]^ Based on the above analysis, a thin and uniform Li_2_S layer with good mechanical strength was successfully constructed on Li surface via a facile CVD method.

### 2.2 Electrochemical and Mechanical Stability of the Li/Li_2_S/LGPS Interface

**Figure 3:**
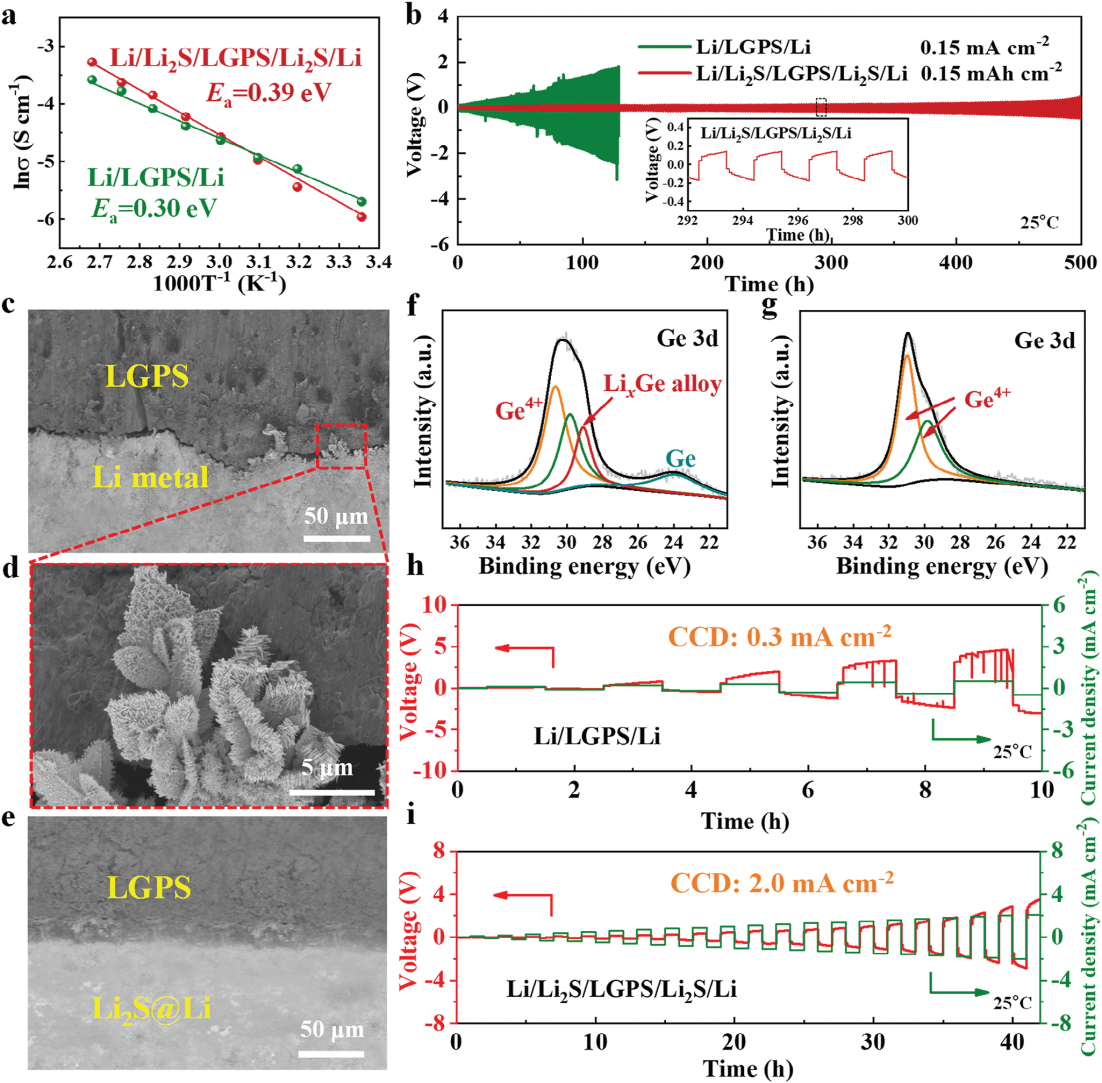
Symmetric cell tests: a) Arrhenius plots of the Li/Li_2_S/LGPS/Li_2_S/Li and Li/LGPS/Li cells; b) galvanostatic Li plating/stripping profiles of the Li/LGPS/Li (green) and Li/Li_2_S/LGPS/Li_2_S/Li (red) cells at 0.15 mA cm^−2^ and 0.15 mAh cm^−2^ (Inset is the enlarged galvanostatic Li plating/stripping profile of the Li/Li_2_S/LGPS/Li_2_S/Li cell.); c) cross‐section SEM image of the Li/LGPS interface without a Li_2_S layer after cycling 50 h in the Li/LGPS/Li cell and d) the enlarged SEM image marked by red box in Figure 3c; e) cross‐section SEM image of the Li/LGPS interface with a Li_2_S layer after cycling 150 h in the Li/Li_2_S/LGPS/Li_2_S/Li cell; f, g) Ge 3d spectra of the LGPS surface in the Li/LGPS/Li and Li/Li_2_S/LGPS/Li_2_S/Li cells after cycling; h, i) CCD tests of the Li/LGPS/Li and Li/Li_2_S/LGPS/Li_2_S/Li cells.

To elucidate the chemical compatibility of Li_2_S with LGPS, XRD, and Raman experiments were performed on a mixture of commercial Li_2_S and LGPS after ball milling treatment. As presented in Figure S4 (Supporting Information), the diffraction peaks of the Li_2_S/LGPS mixture correspond well to the original Li_2_S and LGPS phases, which indicates that Li_2_S remains stable with LGPS, thus avoiding additional interfacial side reactions. To further clarify the effect of the Li_2_S layer on interfacial Li‐ion transport, the temperature‐dependent interfacial impedances of the Li/Li_2_S/LGPS/Li_2_S/Li and Li/LGPS/Li symmetric cells were investigated from 25 to 100 °C by electrochemical impedance spectroscopy (EIS) tests. Based on the Arrhenius equation, the Li‐ion activation energies (*E*_a_) at the interface of Li/LGPS and Li/Li_2_S/LGPS were calculated to be 0.30 and 0.39 eV, respectively (**Figure** **3**a). The small *E*_a_ difference suggests that the in situ formed Li_2_S layer between Li and LGPS exerts a less obstruction effect on Li‐ion migration.

Galvanostatic Li plating/stripping experiments of the Li/LGPS/Li and Li/Li_2_S/LGPS/Li_2_S/Li cells were conducted to depict the role of the Li_2_S layer in promoting the interface stability between Li and LGPS. As displayed in Figure 3b, the Li/LGPS/Li cell exhibited a large overpotential exceeding 1.5 V after cycling for 100 h at 0.15 mA cm^−2^ (0.15 mAh cm^−2^), which indicates that severe side reactions occurred at the interface between Li and LGPS. In stark contrast, after the introduction of the Li_2_S layer, the overpotential of the Li/Li_2_S/LGPS/Li_2_S/Li cell is below 0.2 V after cycling for 300 h, and lower than 0.5 V after further cycling to 500 h under the same conditions. The impedance of the Li/LGPS/Li and Li/Li_2_S/LGPS/Li_2_S/Li cells before and after cycling was analyzed. Figure S5a,b (Supporting Information) show that the Nyquist plots contain an intersection with the X‐axis and two semicircles: the initial intersection at high frequency represents the bulk resistance (R1), and the two semicircles in the middle frequency and low‐frequency regions are assigned to the Li/LGPS interface (R2) and Li‐ion diffusion resistance, respectively.^[^ ^23^ ^]^ The corresponding resistances of the Li/LGPS/Li and Li/Li_2_S/LGPS/Li_2_S/Li cells were obtained by fitting the equivalent circuit and presented in Figure S5c,d (Supporting Information). The initial R1 and R2 values of the Li/Li_2_S/LGPS/Li_2_S/Li cell are 52.8 and 97.8 Ω, which is higher than those of the Li/LGPS/Li cell (R1: 32.3 Ω, R2: 18.2 Ω). The increased resistance can be attributed to the introduction of the Li_2_S layer, whose ionic conductivity was calculated to be 6.6 × 10^−6^ S cm^−1^ (Note S1, Supporting Information). The bulk and interfacial resistance of the Li/LGPS/Li cell dramatically increased to 1500 and 3000 Ω after cycling 50 h, respectively. In contrast, owing to the good protection of Li_2_S layer, the Li/Li_2_S/LGPS/Li_2_S/Li cell exhibited a much smaller bulk (85.7 Ω) and interfacial (278.7 Ω) resistance after cycling for 150 h. The interfacial stability was also illustrated by demonstrating the increase in impedance versus chemical aging time in symmetrical Li/LGPS/Li and Li/Li_2_S/LGPS/Li_2_S/Li cells. The interphase formation between Li and LGPS was observed using time‐resolved impedance spectroscopy. Impedance spectra recorded over the course of 100 h are shown in Figure S6a (Supporting Information), a strong increase of the overall resistance of the Li/LGPS/Li cell is observed. As a comparison, Li_2_S@Li could maintain good interfacial stability with LGPS, and the assembled Li/Li_2_S/LGPS/Li_2_S/Li cell exhibits lower impedance changes after 100 h of aging (Figure S6b, Supporting Information).

The cross‐sections of the symmetric cells were observed after cycling. As displayed in **Figure** **3**c–e, obvious cracks and coarse lithium dendrites appeared at the interface between the pristine Li and LGPS (Figure 3c,d), while the Li_2_S@Li maintained intimate contact with LGPS (Figure 3e). The chemical composition of the LGPS near the Li side after cycling was further analyzed by XPS tests, as shown in Figure 3f,g. In the Ge 3d spectrum of Li/LGPS/Li (Figure 3f), compared to the original Ge 3d spectrum of LGPS (Figure S7, Supporting Information), the peaks appearing at 31 and 29.8 eV refer to the Ge^4+^ of LGPS. Meanwhile, another two new peaks located at 24 and 29 eV could also be found, which correspond to the Ge and Li*_x_* Ge alloy, respectively, indicating that LGPS was severely reduced by Li metal.^[^ ^40^ ^]^ Since Ge and Li*_x_* Ge are good electron conductors that allow the smooth passage of electrons, continuous LGPS decomposition will be triggered in the following cycles. From the Ge 3d spectrum of Li/Li_2_S/LGPS/Li_2_S/Li that is presented in Figure 3g, except for the peaks of the LGPS, there are no remaining peaks, from which it can be inferred that the structure of LGPS was well preserved.

The critical current density (CCD) reflects the maximum Li‐ion migration and charge transfer capacity that can be tolerated, and is used to assess the stability of the Li/LGPS interface and the capability to resist Li dendrite growth.^[^ ^41^ ^]^ As presented in Figure 3h, the assembled Li/LGPS/Li cell exhibits a CCD of 0.3 mA cm^−2^, while the CCD of the symmetric cell with a Li_2_S layer reaches up to 2.0 mA cm^−2^ (Figure 3i). The significant improvement in CCD could be mainly ascribed to the Li_2_S interlayer that prevented the severe side reactions between Li and LGPS.

### 2.3 All‐Solid‐State Li–S Battery with an Artificial Li_2_S SEI Layer

**Figure 4:**
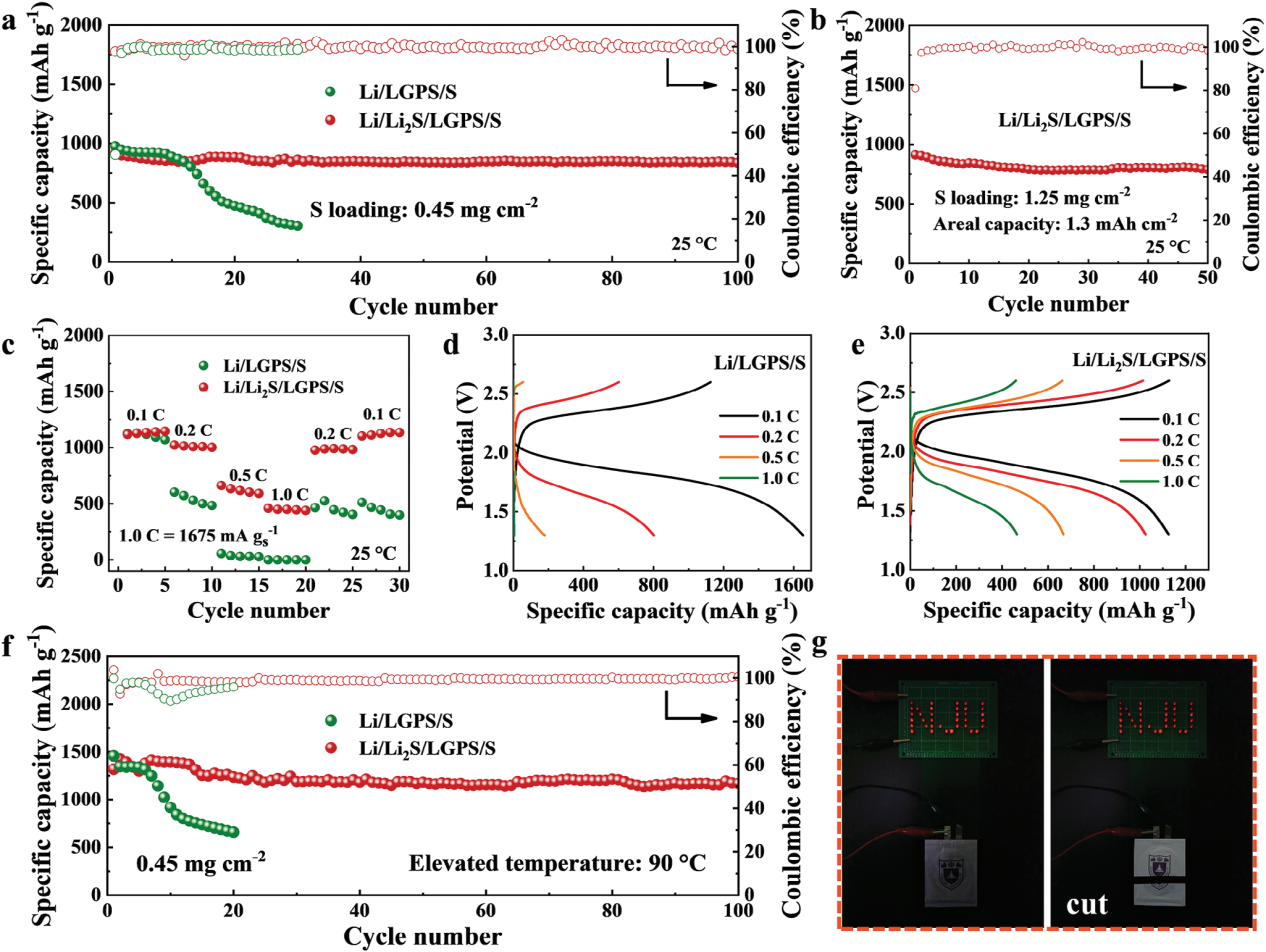
All‐solid‐state Li‐S battery tests: a) Cycling performance of the Li/LGPS/S and Li/Li_2_S/LGPS/S cells under the condition of 0.2 mA cm^−2^ and 0.45 mg cm^−2^; b) cycling performance of the Li/Li_2_S/LGPS/S cell with a higher S loading of 1.25 mg cm^−2^; c) rate performance of the Li/LGPS/S and Li/Li_2_S/LGPS/S cells and the first discharge/charge curves of (d) Li/LGPS/S and (e) Li/Li_2_S/LGPS/S at various current densities ranging from 0.1C to 1.0C; f) cycling performance of the Li/LGPS/S and Li/Li_2_S/LGPS/S cells under harsh condition of 90 °C; g) illustration of the Li/Li_2_S/LGPS/S pouch cell powering an LED light.

To better gain an insight of the effectiveness of Li_2_S as an artificial SEI between Li and LGPS, the ASSLSBs were assembled with sulfur cathode (the content of active material in sulfur cathode is ≈25%, Figure S8, Supporting Information), Li anode, and LGPS electrolyte. The specific reaction process of the Li/Li_2_S/LGPS/S cell was revealed by cyclic‐voltammetry test. As presented in Figure S9 (Supporting Information), during the cathodic scans, the peak at ≈2.5 V can be ascribed to the conversion of S to Li_2_S. During the anodic scans, the peak at ≈1.7 V is attributed to the reversible conversion of Li_2_S to form S.^[^ ^42^ ^]^ **Figure** **4**a shows the cycling performance of the Li/LGPS/S and Li/Li_2_S/LGPS/S cells. At the current density of 0.2 mA cm^−2^ and a S loading of 0.45 mg cm^−2^, the Li/LGPS/S cell experienced rapid capacity decay from the initial capacity of 976.6 to 304.7 mAh g^−1^ after 30 cycles, while the Li/Li_2_S/LGPS/S cell delivered an initial reversible capacity of 921.1 mAh g^−1^, and remained at 836.8 mAh g^−1^ after 100 cycles, which corresponds to a capacity retention of 90.8%. The corresponding discharge/charge curves of the Li/LGPS/S and Li/Li_2_S/LGPS/S cells at different cycles were compared and presented in Figure S10 (Supporting Information), where the Li/Li_2_S/LGPS/S cell presents better cycling stability and smaller voltage polarization. Notably, the Li/Li_2_S/LGPS/S cell exhibits an average discharge voltage of 1.8 V (obtained by integrating the discharge curve), which is 0.6 V higher than the Li/In/LGPS/S cell under the same conditions (Figure S11, Supporting Information), indicating that higher energy density could be achieved by using a Li_2_S modified Li anode. The performance of the Li/Li_2_S/LGPS/S cell was further evaluated at a higher S loading of 1.25 mg cm^−2^ (1.3 mAh cm^−2^). The cell could deliver a reversible capacity of 912.2 mAh g^−1^ at 0.15 mA cm^−2^, and maintained at 785.7 mAh g^−1^ with a capacity retention of 86.1% after 50 cycles (Figure 4b).

Lithium dendrite growth and LGPS decomposition will be more severe under high current conditions. As shown in Figure 4c, rate experiments were carried out at different current densities. The Li/Li_2_S/LGPS/S cell delivered reversible capacities of 1117.1, 1022.9, 662.9, and 460 mAh g^−1^ at 0.1C, 0.2C, 0.5C, and 1.0C, respectively. When the current density returns to 0.1C, a high specific capacity of 1102.9 mAh g^−1^ could be recovered. The Li/LGPS/S cell exhibited an initial reversible capacity of 1125.6 mAh g^−1^ at 0.1C, which is comparable to the Li/Li_2_S/LGPS/S cell. With increased rate, the Li/LGPS/S cell showed much lower specific capacities of 602.3 and 55.8 mAh g^−1^ at 0.2C and 0.5C, respectively. At a higher rate of 1.0C, almost no capacity could be released. Even when the current density came back to 0.1C, the capacity could not be restored and constantly decayed in the following cycles instead.

Figure 4d,e shows the first discharge/charge curves of the Li/LGPS/S and Li/Li_2_S/LGPS/S cells at 0.1C, 0.2C, 0.5C and 1.0C, respectively. Higher irreversible capacities existed in Li/LGPS/S than Li/Li_2_S/LGPS/S at different rates, which could be attributed to the severe LGPS decomposition that provided a large part of the pseudo‐capacities. Moreover, due to the accumulation of interfacial by‐products between Li and LGPS, the Li/LGPS/S cell exhibits greater voltage polarization than Li/Li_2_S/LGPS/S. The above results further demonstrate that the interface compatibility between Li and LGPS could be effectively promoted by the Li_2_S layer.

The cycling stability of the Li/Li_2_S/LGPS/S cell were also evaluated under high temperature conditions. Generally, raising temperature is conducive to accelerating the reaction kinetics of the active S material, thereby reducing voltage polarization and providing higher reversible capacities. Meanwhile, the interface stability between Li and LGPS will confront tougher challenges, since electrolyte decomposition will be simultaneously aggravated at high temperatures. As presented in Figure S12a (Supporting Information), the Li/Li_2_S/LGPS/S cell delivered an initial reversible capacity of 1046.4 mAh g^−1^ at 60 °C and a superb cycling stability for over 120 cycles with a decent capacity retention of 79.0%, which indicates that the Li/Li_2_S/LGPS/S cell has great potential for application in various scenarios. Due to serious interfacial side reactions between Li and LGPS, the Li/LGPS/S cell experienced significant capacity fade at 60 °C after 10 cycles, with a much lower capacity of 330.6 mAh g^−1^ remained (Figure S12b, Supporting Information). Cycling tests of the Li/Li_2_S/LGPS/S cell were further performed at 90 °C. As shown in Figure 4f, a higher initial reversible capacity of 1318.8 mAh g^−1^ could be obtained and maintained at 1168.8 mAh g^−1^ after 100 cycles, which corresponds to a high capacity retention of 88.6% (vs 45.3% after 20 cycles for the Li/LGPS/S cell). The corresponding discharge/charge curves of the Li/Li_2_S/LGPS/S cell at 90 °C are presented in Figure S13 (Supporting Information). Compared to ambient conditions, the cell exhibits better reversibility and less polarization at 90 °C. The above performances are better than most of the literature that LGPS was employed as the electrolyte for ASSLSBs,^[^ ^29^ ^,^ ^43^ ^–^ ^49^ ^]^ as summarized in Figure S14 (Supporting Information). Meanwhile, the application of Li_2_S as an artificial SEI is also very competitive compared to the reported artificial SEIs used to improve the interfacial stability between Li and electrolyte in ASSLSBs,^[^ ^23^, ^25^, ^47^, ^50^, ^51^, ^52^ ^]^ as summarized and presented in Table S1 (Supporting Information). In addition, a Li/Li_2_S/LGPS/S pouch cell was assembled, and the battery was able to power a LED light of the “NJU” logo easily. Even after being cut a part in the air, the battery could still be well‐running (Figure 4g).

### 2.4 Investigation of Interfacial Impedance Evolution Behavior

**Figure 5:**
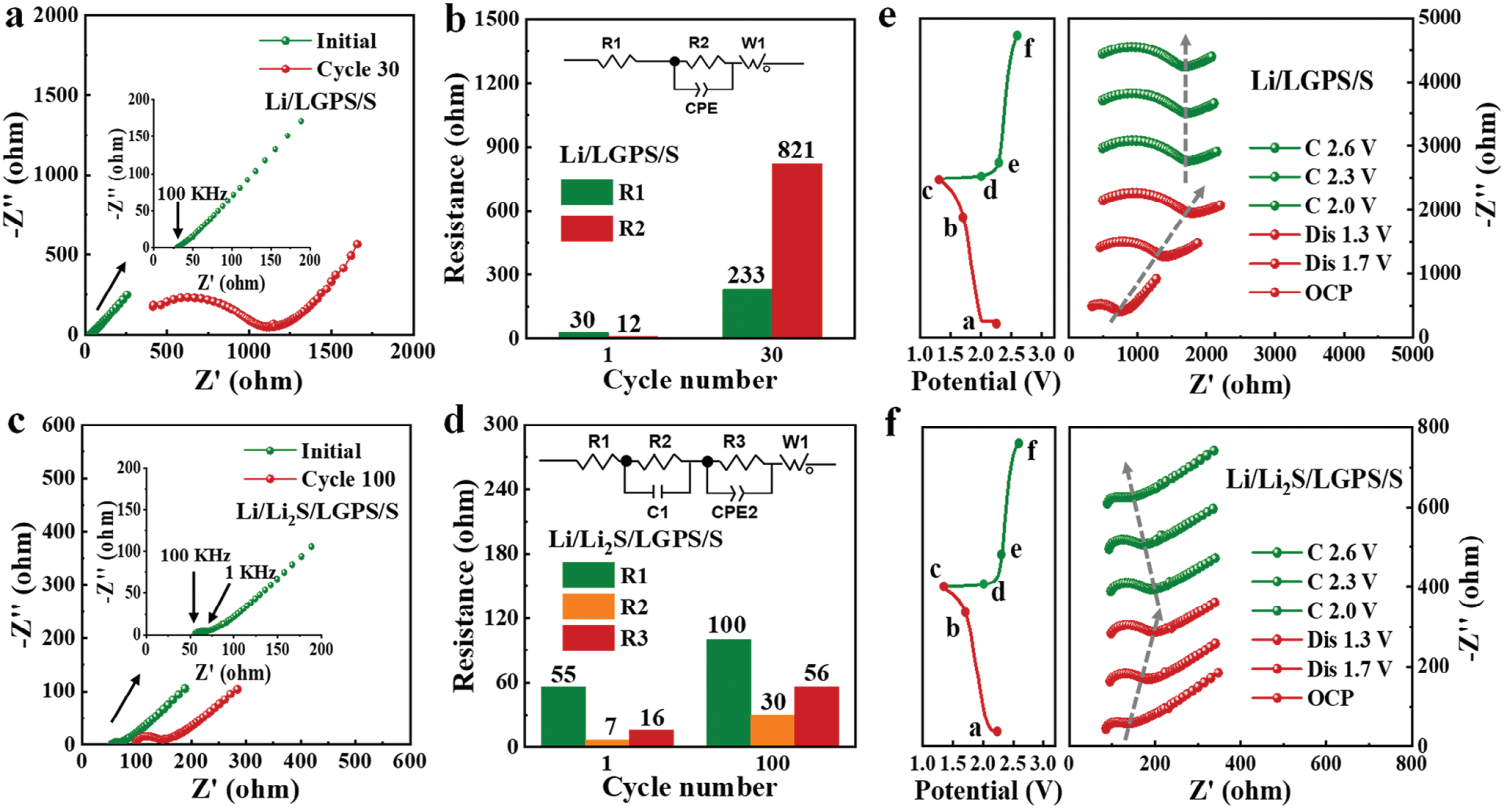
Interface evolution behavior between Li and LGPS with or without a Li_2_S protective layer: EIS results and the corresponding equivalent circuit and resistances of a,b) Li/LGPS/S and c,d) Li/Li_2_S/LGPS/S before and after cycling; e,f) in situ EIS results of the Li/LGPS/S and Li/Li_2_S/LGPS/S cells collected at different discharge/charge states after ten prior cycles.

The impedance changes of the Li/LGPS/S and Li/Li_2_S/LGPS/S cells before and after cycling were analyzed by the EIS tests. **Figure** **5**a shows the Nyquist plots of the Li/LGPS/S cell collected at the original state and cycling for 30 times. Similarly, the intersection with the X‐axis represents the bulk resistance (R1), and the appeared semicircle can be assigned to the interface resistance of Li/LGPS and LGPS/S (R2), while the inclined line can be attributed to the Warburg‐type resistance. The plots were fitted via the equivalent circuit, as shown in Figure 5b. The values of R1 and R2 are 30 and 12 Ω at the original state, which dramatically increased to 233 and 821 Ω after 30 cycles. From the EIS result of the Li/Li_2_S/LGPS/S cell (Figure 5c), a new semicircle appeared at the frequency ranging from 1 to 100 KHz, which corresponds to the interface resistance of Li_2_S/LGPS (R3). The interface resistance between Li and Li_2_S can be neglected due to in situ generation of Li_2_S on Li surface. After cycling for 100 times, the values of R1, R2, and R3 changed from 55, 7, and 16 to 100, 30, and 56 Ω, respectively (Figure 5d).

In situ EIS experiments were further carried out to reveal the impedance evolution of Li/LGPS/S and Li/Li_2_S/LGPS/S cells. In order to visualize the differences in impedance changes more intuitively, the Li/LGPS/S and Li/Li_2_S/LGPS/S cells were first cycled for 10 times and then subjected to in situ EIS measurements. As displayed in Figure 5e, resistance changes were clearly observed in Li/LGPS/S during discharge/charge process. From the fitted values of the EIS results, shown in Figure S15a (Supporting Information), the interface resistance of the Li/LGPS/S cell increased dramatically from 566.9 to 1427 Ω when discharged to 1.3 V, and the high interfacial impedance is maintained stable until the cut‐off potential is reached, indicating that the Li/LGPS interface is severely damaged. In stark contrast, the interfacial impedance of the Li/Li_2_S/LGPS/S cell gradually increases during discharge and then decreases during charge with negligible variation, which implies that the Li/Li_2_S/LGPS/S cell has excellent reversibility. (Figure 5f; Figure S15b, Supporting Information) The above analysis reaffirmed that the presence of the Li_2_S layer could efficiently maintain the interface stability between Li and LGPS.

## 3 Conclusion

In summary, we demonstrated the feasibility of Li_2_S as an artificial SEI to modify the interface stability between Li metal anode and LGPS electrolyte, and successfully constructed a thin and homogeneous Li_2_S layer on Li surface via a facile CVD method. With the robust Li_2_S protective layer, the symmetric cells exhibited more stable Li‐plating/stripping overpotential (< 0.5 V) over 500 h at 0.15 mA cm^−2^ and higher CCD of 2.0 mA cm^−2^. The ASSLSBs of Li/Li_2_S/LGPS/S delivered a reversible capacity of 921.1 mAh g^−1^ at 0.2 mA cm^−2^ with a S loading of 0.45 mg cm^−2^ and operated steadily for 100 cycles with a greatly improved capacity retention of 90.8%. And a decent capacity retention of 86.1% was achieved at 0.15 mA cm^−2^ with a higher S loading of 1.25 mg cm^−2^ (1.3 mAh cm^−2^) after 50 cycles. Under the harsh conditions of 60 and 90 °C, the Li/Li_2_S/LGPS/S cell exhibited higher initial reversible capacities of 1046.4 and 1318.8 mAh g^−1^ with capacity retentions of 79.0% and 88.6% after 120 and 100 cycles, respectively. We believe that such a simple, economical and environmentally friendly interface modification strategy will not only provide new sights for the performance improvement of ASSLSBs, but also open a new avenue for the development of designing high‐performance all‐solid‐state lithium batteries based on sulfide electrolytes.

## 4 Experimental Section

### Materials Preparation

The sulfur powder (99%) was obtained from Alfa Aesar. The multi‐walled carbon nanotubes (MWCNTs, > 95%) and Li_2_S powder (99.9%) were obtained from Macklin. The LGPS was purchased from Hefei Kejing Material Technology Co. Ltd., whose ionic conductivity was calculated to be ≈1.9 × 10^−3^ S cm^−1^ (Note S2, Supporting Information), and the corresponding electrochemical impedance spectroscopy (EIS) and scanning electron microscopy (SEM) of the LGPS are presented in Figure S16 (Supporting Information). All materials were directly used without further purification.

### The Preparation of Li_2_S Layer on the Li Metal Surface

Li foil was first scraped with a knife to remove the surface impurities and subsequently rolled with a metal rod. The obtained Li sheet was then punched into Li discs with a diameter of 10 mm. Five Li discs were attached to the inner wall of a 100 ml thick‐walled reaction flask with 0.5 g of sulfur powder, then the flask was sealed and heated at 160 °C for 3 h. To avoid the presence of residual sulfur on the Li metal surface, the treated Li discs were heated for another 6 h. All operations were conducted in an Ar‐filled glove box where oxygen and water content are below 0.01 ppm.

### Preparation of Sulfur Cathode

The sulfur cathode was prepared according to the method that have been previously reported.^[^ ^40^ ^]^ Briefly, S and MWCNTs were homogeneously mixed in a mass ratio of 7:3. Then the mixture was sealed in a glass vial and heated at 155 °C for 5 h under Ar atmosphere. The obtained powder was ground for another 30 min and heated at 200 °C for 2 h to obtain the S@MWCNTs precursor. The S@MWCNTs, MWCNTs, and LGPS were ball milled at 500 rpm for 6 h under Ar atmosphere according to the mass ratio of 3:1.5:3.5 to obtain the final sulfur cathode.

### Cell Assembly

For symmetric cells, 100 mg of LGPS was weighed into a poly(ether‐ether‐ketone) (PEEK) mold with an internal diameter of 10 mm and held under 280 MPa for 5 min. The untreated Li discs and the Li_2_S modified Li discs were then placed on both sides of the pressed electrolyte and 80 MPa pressure was applied to ensure a tight contact. For the ASSLSBs assembly, 100 mg of LGPS was weighed into the PEEK mold and held under 280 MPa for 5 min. The prepared sulfur cathode was evenly distributed on one side of the electrolyte and pressed under 300 MPa for another 5 min. The untreated Li disc or the Li_2_S modified Li disc was placed on the other side of the electrolyte. Al and Cu foils were used as the collectors at the cathode and anode sides, respectively. The assembled cell was operated under pressure of 80 MPa by a stainless‐steel frame. As for the assembly of all‐solid‐state Li–S pouch cell, first, the sulfur cathode was thoroughly mixed with polytetrafluoroethylene with a mass ratio of 99:1 and ground with an agate mortar until a dough was formed. Then the dough was roll‐pressed into a sheet with desired thickness to obtain the cathode film. The LGPS electrolyte film was fabricated following a similar process. Finally, the sulfur cathode layer, LGPS membranes, and a Li_2_S modified Li sheet were pressed together and vacuum sealed in aluminum plastic film. Nickel and aluminum metal tabs were welded to anode and cathode side as collectors, respectively.

### Electrochemical Tests

The galvanostatic discharge/charge tests for symmetric and full cells were performed on the Neware battery test system; cyclic‐voltammetry (CV) test was conducted on the CHI with a scan rate of 0.1 mV s^−1^; EIS tests were performed on the Solartron with an applied frequency range of 0.1–10^6^ Hz and an amplitude of 5 mV; for high temperature tests, the cells were placed and operated in a thermostat under the specific temperature. The voltage range was set to 1.3–2.6 V, and all calculations were based on the mass of S.

### Materials Characterization

X‐ray diffraction (XRD) analysis was carried out to analyze the phase structure of Li_2_S layer by employing a Bruker D8 advanced diffractometer with Cu–Kα radiation (*λ* = 1.5406 Å) at a scan rate of 0.02° s^−1^. X‐ray photoelectron spectroscopy (XPS) test was performed on PHI 5000 VersaProbe‐II. The morphology and thickness observations of the Li_2_S layer was conducted by SEM (Hitachi SU8010) and the corresponding composition mapping was obtained by the equipped energy dispersive X‐ray spectrometry (EDS). The cross‐sectional microstructure of the Li_2_S layer was observed by focused ion/electron dual‐beam electron microscopy (Helios G4 CX). The microstructure and composition were further characterized by transmission electron microscopy (TEM, FEI TF20), and the selected area electron diffraction (SAED) pattern was collected from a Gatan charge‐coupled device camera. Atomic force microscopy (AFM, Bruker Dimension ICON) and RTESP‐525 tip were used to analyze the surface smoothness and Young's modulus of the Li_2_S layer. Raman spectra were carried out on a confocal Raman microscope (inVia, Renishaw) with an excitation wavelength of 633 nm. Thermogravimetry (TG) test was conducted on an SDT Q600 TA instrument under Ar atmosphere. The temperature range was set to 25–600 °C and the heating rate was 10 °C min^−1^.

## Conflict of Interest

The authors declare no conflict of interest.

## Supporting information

Supporting Information

## Data Availability

The data that support the findings of this study are available from the corresponding author upon reasonable request.
