# Supplementary material for: Integrating Lithium Sulfide as a Single Ionic Conductor Interphase for Stable All‐Solid‐State Lithium–Sulfur Batteries
Source: Adv Sci (Weinh). 2024 Apr 23;11(25):2308604. doi: 10.1002/advs.202308604 (PMC11220677; doi:10.1002/advs.202308604)
Supplement: Supplementary file 1 — Supporting Information [file ADVS-11-2308604-s001.docx]

Supporting Information

**Integrating Lithium Sulfide as a Single Ionic Conductor Interphase for Stable All-Solid-State Lithium-Sulfur Batteries**

*Xin Wu, Hui Pan, Menghang Zhang, Hanyun Zhong, Zhenjie Zhang, Wei Li, Xinyi Sun, Xiaowei Mu, Shaochun Tang*^*^*, Ping He*^*^*, and Haoshen Zhou*


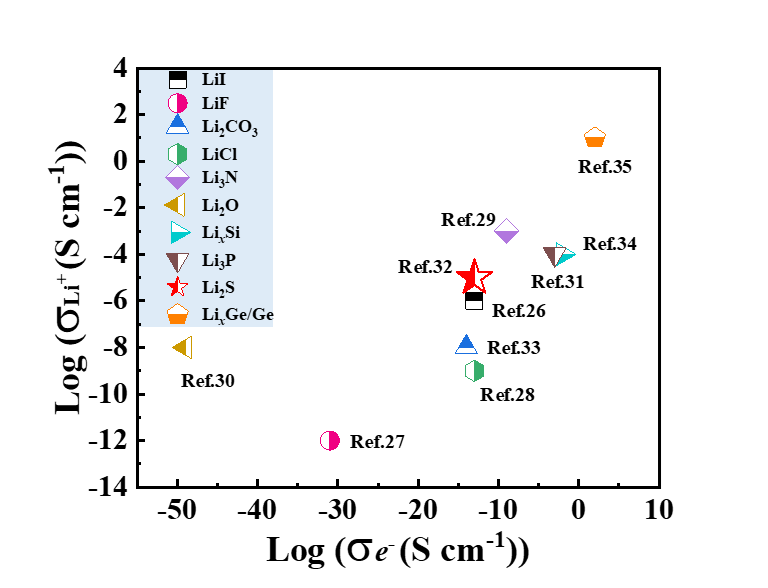


Figure S1. Summary of ionic and electronic conductivity of common SEI components reported in the literature.


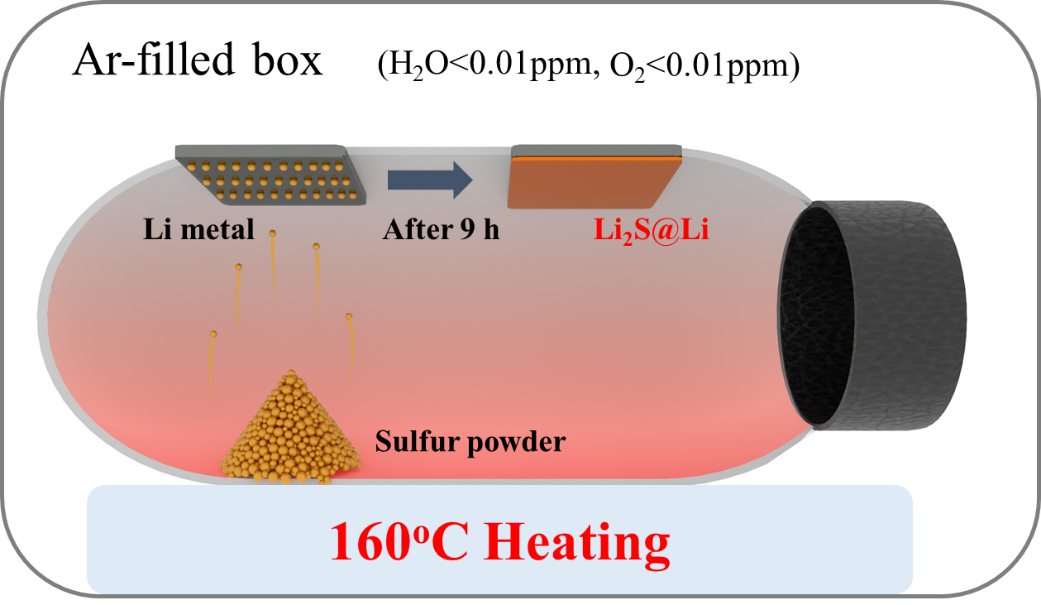


Figure S2. Schematic of Li_2_S@Li prepared by chemical vapor deposition.


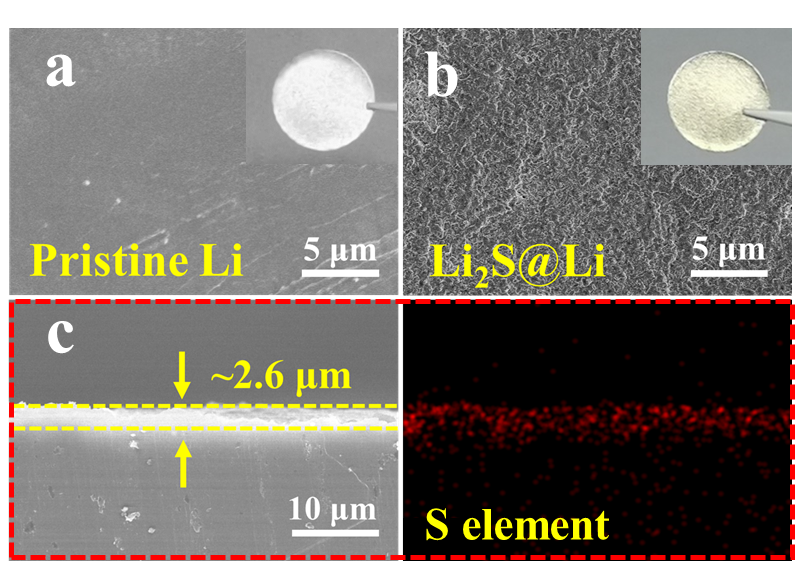


**Figure S3.** a, b) SEM images of Li metal before and after Li_2_S layer coating (Inset is the corresponding digital photo.); c) cross-section SEM image of the Li_2_S@Li and the corresponding EDS image.

Figure S4. a) The XRD pattern and b) Raman spectra of pristine LGPS (green line), pristine Li_2_S (orange line), and the mixture of Li_2_S and LGPS with the weight ratio of 1:1. (red line). No peak change occurs for the mixture of Li_2_S and LGPS after ball-milling for 3h, suggesting that Li_2_S is chemically stable with LGPS.

Figure S5. a, b) Nyquist plots of the Li/Li_2_S/LGPS/Li_2_S/Li and Li/LGPS/Li cells before and after cycling and the corresponding equivalent circuit; c, d) The fitted impedance values of the Li/Li_2_S/LGPS/Li_2_S/Li and Li/LGPS/Li cells before and after cycling.

Figure S6. a, b) Nyquist plots showing the impedance evolution *vs.* chemical aging time in symmetrical Li/LGPS/Li and Li/Li_2_S/LGPS/Li_2_S/Li cells. The increasing impedance with time shows an increase of the interfacial resistance, implying a rapid decomposition of the LGPS electrolyte when in contact with Li.

Figure S7. XPS spectrum of Ge 3d for the pristine LGPS.

Figure S8. TG curve for the sulfur cathode at a heating rate of 10°C min^−1^ under Ar flow. S will sublimate when heated to a certain temperature, and the mass loss corresponds to the content of S.

Figure S9. The CV curves of the Li/Li_2_S/LGPS/S cell at a scanning rate of 0.1 mV s^-1^.

Figure S10. The discharge/charge curves of a) Li/LGPS/S and (b) Li/Li_2_S/LGPS/S cells at 25°C under different cycles.

Figure S11. The comparison of the discharge/charge curves between the Li/Li_2_S/LGPS/S and Li/In/LGPS/S cells under the same testing conditions.

Figure S12. Cycling performance of (a) Li/Li_2_S/LGPS/S and (b) Li/LGPS/S cells at 0.2 mA cm^−2^ and 60°C with a S loading of 0.45 mg cm^-2^.

Figure S13. The discharge/charge curves of the Li/Li_2_S/LGPS/S cell at 90°C under different cycles.


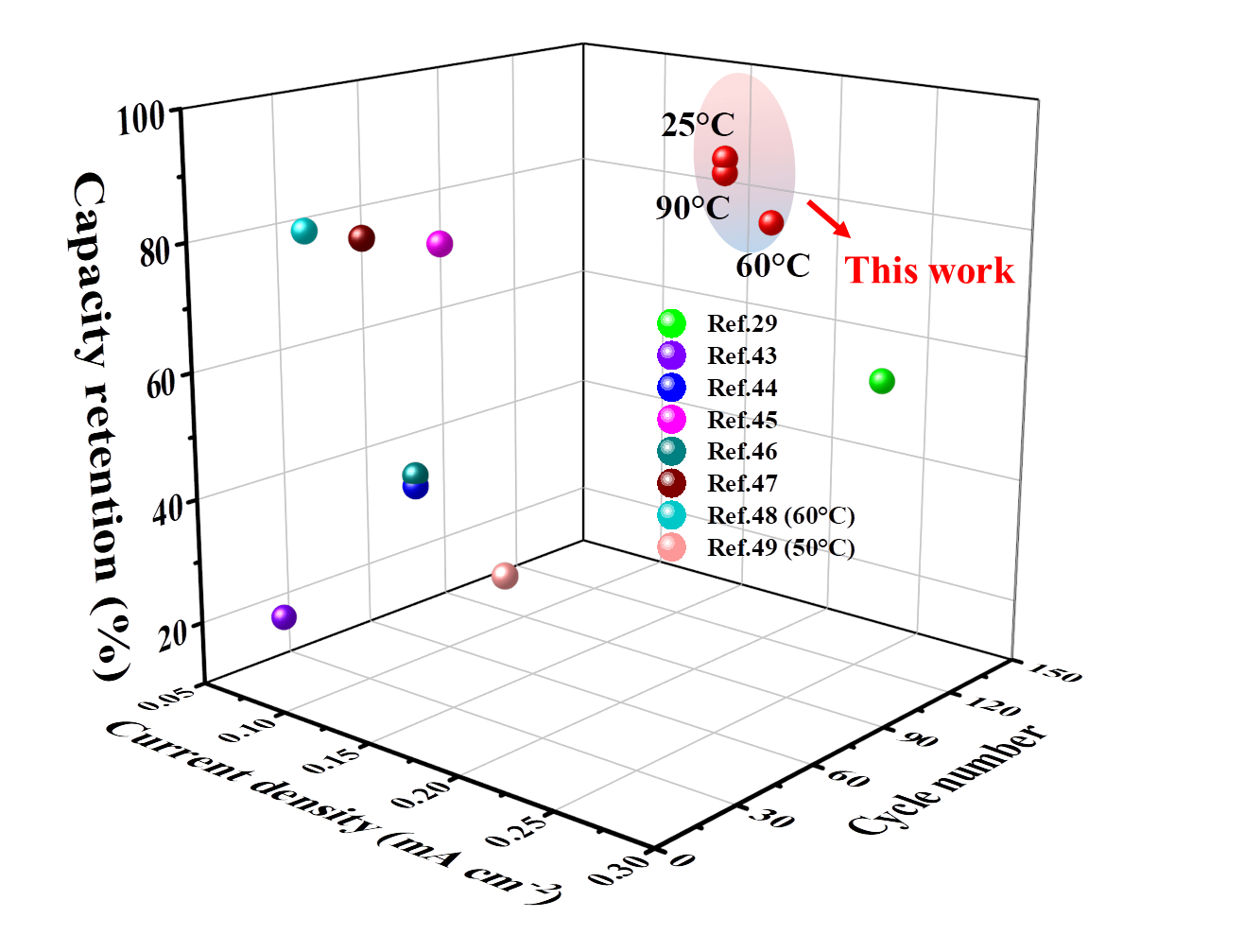


Figure S14. The performance comparison with previously reported ASSLSBs using LGPS as an electrolyte.

Figure S15. The corresponding fitted values of the interface resistance at different discharge/charge states shown in Figure 5e and Figure 5f.

Figure S16. a) Nyquist plot and b) SEM image of LGPS.

Note:

The ionic conductivity can be obtained by the following formulas:

$$\boldsymbol{R}\mathbf{=}\frac{\boldsymbol{L}}{\boldsymbol{\sigma A}} \left( \mathbf{1} \right)$$

$$\boldsymbol{\sigma=}\frac{\boldsymbol{L}}{\boldsymbol{AR}}\mathbf{(2)}$$

Where the $\sigma$ represents the ionic conductivity, R represents the bulk resistance, and L and A represent the thickness and area, respectively.

S1: Estimation of the ionic conductivity of the prepared Li_2_S layer

By comparing the resistance of the Li/LGPS/Li and Li/Li_2_S/LGPS/Li_2_S/Li symmetrical cells, the bulk resistance of the Li_2_S layer can be approximately calculated as follows: (Data is obtained from Figure S5a and Figure S5b).

$$\boldsymbol{R}_{\boldsymbol{Li}_{\mathbf{2}}\boldsymbol{S}}\boldsymbol{\approx}\frac{\boldsymbol{R}_{\mathbf{Li/}\boldsymbol{Li}_{\mathbf{2}}\mathbf{S/LGPS/}\boldsymbol{Li}_{\mathbf{2}}\mathbf{S/Li}}\mathbf{-}\boldsymbol{R}_{\mathbf{Li/LGPS/Li}}}{\mathbf{2}}$$

$$\boldsymbol{R}_{\boldsymbol{Li}_{\mathbf{2}}\boldsymbol{S}}\boldsymbol{\approx}\frac{\mathbf{150.6}\boldsymbol{\Omega}\mathbf{-50.5}\boldsymbol{\Omega}}{\mathbf{2}}\mathbf{=50.1}\boldsymbol{\Omega}$$

The thickness of the Li_2_S layer is obtained from Figure 2f (~2.6 μm), and the area is 0.785 cm^2^.

$$\boldsymbol{\sigma}_{\boldsymbol{Li}_{\mathbf{2}}\boldsymbol{S}}\mathbf{=}\frac{\boldsymbol{2.6\times}\mathbf{10}^{\mathbf{-4}}\boldsymbol{cm}}{\mathbf{0.785}\boldsymbol{cm}^{\mathbf{2}}\boldsymbol{\times50.1}\boldsymbol{\Omega}}\boldsymbol{=6.6\times}\mathbf{10}^{\mathbf{-6}}\boldsymbol{S} \boldsymbol{cm}^{\mathbf{-1}}$$

S2: Calculation of the ionic conductivity of the obtained LGPS electrolyte

The bulk resistance of LGPS is obtained from Figure S16a (74.2 Ω), and the corresponding thickness and area of the LGPS pellet are 1.1 mm and 0.785 cm^2^, respectively.

$$\boldsymbol{\sigma}_{\boldsymbol{LGPS}}\mathbf{=}\frac{\boldsymbol{1.1\times}\mathbf{10}^{\mathbf{-1}}\boldsymbol{cm}}{\mathbf{0.785}\boldsymbol{cm}^{\mathbf{2}}\boldsymbol{\times74.2}\boldsymbol{\Omega}}\boldsymbol{=1.9\times}\mathbf{10}^{\mathbf{-3}}\boldsymbol{S} \boldsymbol{cm}^{\mathbf{-1}}$$

**Table S1.** Comparison between Li_2_S and artificial SEIs reported for application in ASSLSBs.

| Artificial SEIs | Current density | Cycle number  (n) | Capacity retention (%) | Preparation costs and scalability | Refs. |
| --- | --- | --- | --- | --- | --- |
| LiI | 0.2 mA cm^-2^ | 150 | 80.6 | Good | [23] |
| Li*_x_*Mg/LiF/Polymer layer | 0.26 mA cm^-2^ | 120 | 57.3 | Poor | [25] |
| LiF | 83.5 mA g^-1^ | 50 | 82.6 | Poor | [47] |
| Li_3_N | 160 mA g^-1^ | 120 | 45 | Good | [50] |
| 2H‐MoS_2_ | 0.4 mA cm^-2^ | 200 | 86.4 | Good | [51] |
| LiB skeleton/Ag@C layer | 0.416 mA cm^-2^ | 60 | 82 | Moderate | [52] |
| Li_2_S | 0.2 mA cm^-2^ | 100(25°C)  120(60°C)  100(90°C) | 90.8  79  88.6 | Excellent | This work |
